# Effect of preoperative predigested formula vs. polymeric formula on bowel function recovery after definitive surgery for small intestinal entero-atmospheric fistula in patients with chyme reinfusion

**DOI:** 10.3389/fnut.2022.923191

**Published:** 2022-10-21

**Authors:** Weiliang Tian, Zheng Yao, Xin Xu, Shikun Luo, Risheng Zhao

**Affiliations:** ^1^Department of General Surgery, Jinling Hospital, Nanjing, China; ^2^Department of General Surgery, Jiangning Hospital, Nanjing, China

**Keywords:** outcomes, surgery, postoperative ileus, polymeric formula, predigested formula

## Abstract

**Purpose:**

The purpose of this study is to compare the effect of preoperative predigested formula vs. polymeric formula on bowel function recovery following definitive surgery (DS) for small intestinal enteroatmospheric fistula (EAF).

**Methods:**

In this retrospective study, from January 2005 to December 2019, the patients with small intestinal EAF and receiving a DS were enrolled. During the preoperative treatment, each patient received enteral nutrition *via* nasojejunal feeding and chyme reinfusion. The enrolled subjects were classified into two groups, based on their formula type: polymeric formula and predigested formula. Then, propensity scores matching (PSM) was used to further divide these patients into PSM polymeric formula group or PSM predigested formula group. The clinical characteristics of the groups were analyzed.

**Result:**

A total of 137 patients were finally enrolled, with 72 patients in the polymeric formula group and 65 patients in predigested formula group. The postoperative ileus was manifested in a total of 61 (44.5%) cases, with 27 (37.5%) in the polymeric formula group and 34 (52.3%) in the predigested formula group (*P* = 0.04). It was predicted that the polymeric formula could result in a reduction in postoperative ileus (OR = 0.47; 95% CI: 0.21–0.95; *P* = 0.04). After 1:1 PSM, there were 110 patients included. The postoperative ileus was observed in 47 patients, with 18 (32.7%) in the polymeric formula group and 29 (52.7%) in the predigested formula group (*P* = 0.03). After PSM, the polymeric formula demonstrated a reduction in the incidence of postoperative ileus (OR = 0.42; 95% CI: 0.19–0.92; *P* = 0.03).

**Conclusion:**

Compared with predigested formula, the preoperative polymeric formula appears to be associated with earlier recovery of bowel function after DS for EAF.

## Introduction

Enteroatmospheric fistula (EAF), a particular subset of enterocutaneous fistula (ECF), is defined as communication between the gastrointestinal tract and the atmosphere, without skin or soft tissue surrounding or overlying the opening in the bowel (1). EAF is almost impossible to achieve spontaneous closure (2), and a definitive surgery (DS) with high morbidity is essential (3), in which the incidence of postoperative ileus might be up to 50% (4–6). A typical nutritional strategy consists of enteral nutrition (EN) in conjunction with chyme reinfusion (CR) (5–8).

The predigested formula is more readily absorbed than the polymeric formula, making it easier to achieve nutrition goals when gastrointestinal continuity is established with CR. However, it is reasonable that predigested formula is more fully absorbed in the jejunum, so fewer unabsorbed predigested preparations will reach the ileum than the polymeric formula (9). The essential nutrition for gastrointestinal mucosa stimulates chyme rich in nutrients (10). Consequently, this difference in the absorption rate makes it plausible to hypothesize that this may affect the number of nutrients in the distal chyme, thereby affecting the appearance and function of the terminal small intestinal mucosa, thus impacting the healing time of bowel function after DS.

## Methods

### Study design

This was a retrospective study performed at two tertiary hospitals with more than 2,500 beds. The institutional review board approved the study. All procedures were performed in compliance with relevant guidelines and regulations. Informed consent was obtained from all individuals.

### Grouping

From January 2005 to December 2019, the characteristics of patients receiving a DS for small intestinal EAF were reviewed. Before the DS, EN was given *via* nasojejunal tube during the entire treatment process, for which the energy was calculated according to 30 kcal/kg.

Before 2012, CR was not extensively employed. As a result, predigested formula (Peptison Liquid, Nutricia, Wuxi, China) was used until DS due to the characteristics of easy absorption. After 2012, because of the widespread use of CR, polymeric formula (Nutrison Fiber, Nutricia, Wuxi, China) was widely used.

**Figure 1:**
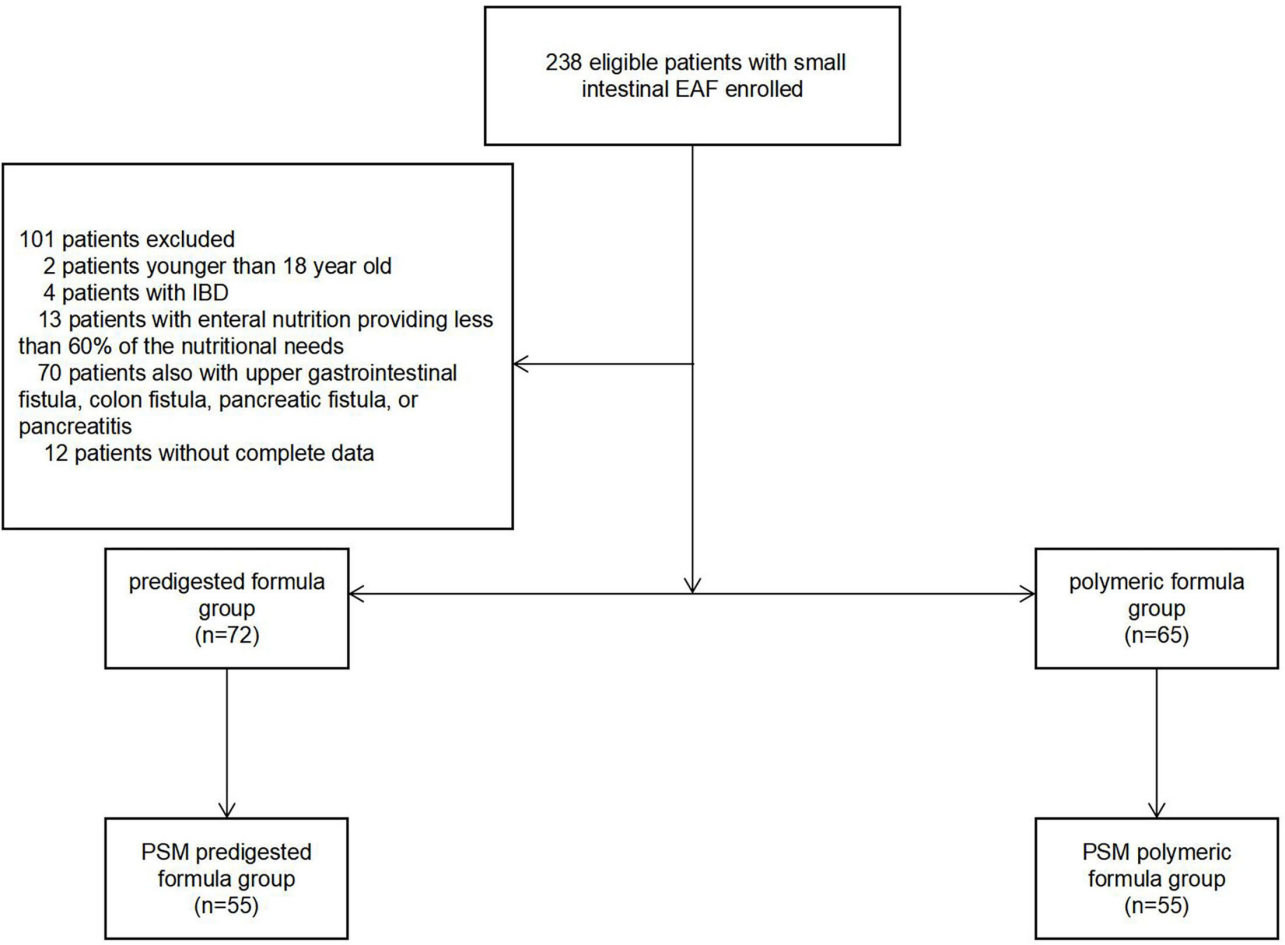
The patients and grouping.

According to the type of formula used, patients were divided into polymeric formula group and predigested formula group (Figure 1). Then, propensity scores matching (PSM) was used to further divide these patients into PSM polymeric formula group or PSM predigested formula group. There was a thorough examination of the groups' clinical features (Figure 1).

### Excluded criterion

The excluded criterion were as follows: (1) patients ≤ 17-year-old; (2) patients with concurrent upper gastrointestinal fistula, colon fistula, pancreatic fistula, or pancreatitis, which may influence the difficulty of the operation; (3) patients with EN providing <60% of the nutritional needs; (4) patients with inflammatory bowel disease (IBD); and (5) patients without complete data.

Patients were followed up to discharge. The primary outcome was postoperative ileus. The secondary outcomes were as follows: (1) defecation time and (2) duration from initial postoperative resume of EN to total EN.

### Treatment of EAF and DS

The treatment of EAF followed the SOWATS treatment guidelines, consisting of the following components: sepsis, optimization of nutritional state, wound care, anatomy (of the leakage), the timing of surgery, and surgical strategy (11). Additionally, the temporary abdominal closure technique (TAC) was used to protect the exposed intestine until a frozen abdomen formed. After forming the frozen abdomen, a planned abdominal hernia was designed *via* stamp skin grafting from the patient's head.

At least 3 months after grafting, DS for EAF was performed. The following are the conclusive surgical criteria. First, C-reactive protein (CRP), white blood cell (WBC), and procalcitonin (PCT) are kept regular for more than 1 month. Second, BMI ≥ 18.0 and normal physical strength are maintained. Third, hemoglobin ≥ 110 g/L. Finally, the interval exceeds 4 months after the first time getting discharged from our institution (5).

Our chief surgeon, Dr. Yunzhao Zhao, MD and Ph.D., performed the DS. During the DS, a lateral-lateral end anastomosis was performed in each fistula using a linear stapler (Pride Medical Inc., Jingjiang, Taizhou, Jiangsu, China). In addition, serosa and muscularis injuries were sutured using a 4–0 absorbable band (Vicryl Plus, Ethicon, Inc., TX, USA). Before anastomosis, the digestive tract was gradually dissociated. In all cases, intra-intestinal splinting was carried out before abdominal closure. In addition to the closure of the fistula(s), hernia repair was also performed for each patient during the DS. Besides, component separation technology was applied and onlay mesh repair was carried out. A Cook Biodesign advanced tissue repair device (Cook Medical Inc., Bloomington, IN, USA) was employed in this process. Negative pressure drainage took place under all incisions. During the postoperative treatment, RBC and human serum Alb were used to maintain patients with hemoglobin (Hb) >100 g/L and/or albumin (Alb) >30 g/L, respectively.

### Perioperative treatment

The preoperative discussion focused on the possible difficulties that may arise during the operation and potential solutions were performed 2 days before each operation. Because the adhesions might be severe and cause excessive bleeding during the operation, at 6 p.m. the day before DS, EN was stopped, and preoperative bowel preparation with sodium phosphate was performed. An additional 1,500 ml of intravenous crystalloid was infused overnight to prevent dehydration. At 6 a.m. on the day of DS, a nasogastric tube for decompression was placed. Second-generation cephalosporin was used within 30 min of the DS as a preoperative prophylactic antimicrobial.

After postoperative defecation, the EN with predigested formula began to resume. During the process, minimal EN with a speed of 20 ml/h was initially used. Provided the patient had no gastrointestinal symptoms, such as abdominal distension and diarrhea, the speed of EN was progressively raised from 20 to 80 ml/h over 6 days at a rate of 10 ml/h per day. If gastrointestinal symptoms existed, the increase of EN was slowed down to accommodate the patient's circumstances. Due to the prolonged absence of a regular oral diet, postoperative continuous and stable EN was performed for about 1 month. The liquid diet was gradually resumed. During this process, the necessary intravenous fluids were used to maintain electrolyte balance.

### Definition, data collection, and statistical analysis

A preliminary assessment consisted of calculating the gender, age, and time interval from occurrence to admission of the EAF, as well as recording the etiology at admission. The preoperative Hemoglobin (Hb), Albumin (Alb), body mass index (BMI), the output of fistula, and the area of planned ventral hernia were estimated within 1 week before DS. As part of the surgical procedure, the length from the Treitz ligament to the location of the fistula, length of the small intestine, degree of abdominal adhesion, duration of DS, and intraoperative blood loss were evaluated. The patient's postoperative course record was evaluated concerning red blood cell (RBC) and Alb transfusions within 48 h after DS.

The degree of abdominal adhesion was primarily classified according to Hobson KG (12), which could be assessed simply according to the operation record and was suitable for retrospective study (Degree I = no adhesions; Degree II = minimal adhesions localized to 1 or 2 areas; Degree III = diffuse adhesions, but not extensive; Degree IV = diffuse extensive adhesions, easily lysed; Degree V = diffuse extensive, dense adhesions, difficult to lyse). Adhesions occupying more than half the surgical field in the abdominal cavity were considered “diffuse extensive” in our study. In cases where the intestines did not have a gap at 50% adhesion sites, adhesive lesions were termed “dense adhesions.” If the intestinal damage and bleeding during the dissociation were inevitable, the adhesion was defined as “difficult to lyse.” Postoperative ileus was defined as a longer defecation time than 7 days after DS (13).

All statistical analyses were performed using the Statistical Package for Social Science (SPSS) version 26.0 for Windows (IBM, Analytics, Armonk, NY). Comparing continuous variables between groups was carried out using Student's *t*-test and a Mann–Whitney U-test. Comparing categorical variables was conducted using Fisher's exact test. We investigated confounding variables *via* multivariate Cox regression and logistic regression. The practice of estimating treatment effects with observational data is reduced with the use of a 1:1 PSM. The patients in the PSM groups were matched based on the calculated propensity scores by a regression model with demographic data, fistula characteristics, and characteristics related to DS. A value of 0.05 was chosen as the match tolerance. Statistical significance was defined as a *P* < 0.05.

## Results

### Baseline characteristics and population

Our study encompassed 238 patients with small intestinal EAF, who underwent DS from January 2005 to December 2019. There were two patients ≤ 17-year-old, four patients with IBD, 13 patients with EN providing <60% of the postoperative nutritional needs, 70 patients with concurrent upper gastrointestinal fistula, colon fistula, pancreatic fistula, or pancreatitis, and 12 patients without complete data. A total of 137 patients were finally enrolled in our study. Seventy-two patients were assigned to the polymeric formula group and 65 patients were in predigested formula group. Except for the different time period of treatment, the characteristics were comparable between the two groups (Table 1).

**Table 1:** Patients characteristics.

**Clinical variables**	**Polymeric formulas group** ** (*n* = 72)**	**Predigested formula group** ** (*n* = 65)**	***P***	**PSM polymeric formulas group** ** (*n* = 55)**	**PSM predigested formula group** ** (*n* = 55)**	***P***
Demographic data						
Female, No. (%)	39 (54.2)	32 (49.2)	0.69	30 (54.5)	29 (52.7)	0.85
Age, years; (median,IQR)	45.0 (34.0–54.75)	42.0 (37.0–51.0)	0.98	43.0 (32.0–54.0)	41.0 (37.0–48.0)	0.36
BMI, kg/m^2^, (median,IQR )	19.0 (18.3–20.1)	19.2 (18.5–20.2)	0.17	19.0 (18.3–20.1)	19.4 (18.5–20.3)	0.99
Time period, No. (%)			-			-
Year 2005–Year 2008	-	30 (21.9)		-	26 (23.6)	
Year 2008–Year 2011	-	35 (25.5)		-	29 (26.4)	
Year 2012–Year 2015	32 (23.4)	-		25 (22.7)	-	
Year 2016–Year 2019	40 (29.2)	-		30 (27.3)	-	
Fistula characteristics						
Interval from fistula occurred to admission, day, (median,IQR)	15 (12–19)	16 (14–19)	0.26	15 (13–21)	16 (14–19)	0.89
Distance from treitz ligament to fistula, No. (%)			0.78			0.78
<100 cm	25 (34.8)	22 (33.8)		17 (30.9)	18 (32.7)	
From 100 to 200 cm	33 (45.8)	33 (50.8)		25 (45.5)	27 (49.1)	
>200 cm	14 (19.4)	10 (15.4)		13 (23.6)	10 (18.2)	
Length of small intestine, No. (%)			0.58			0.65
<300 cm	17 (23.6)	18 (27.7)		14 (25.5)	12 (21.8)	
≥300 cm	55 (76.4)	47 (72.3)		41 (74.5)	43 (78.2)	
Duration of CR, month (median, IQR)	5 (4–6)	5(4–6)	0.78	5 (4–6)	5(4–6)	0.89
Duration of enteral nutrition, month (median, IQR)	5 (4–6)	5(4–6)	0.78			
Duration of usage of polymeric formulas, month (median, IQR)	4 (3–4)	-	-	4 (3–4)	-	-
High output, No. (%)	69 (95.8)	63 (96.9)	1.00	65 (100)	65 (100)	1.00
The area of planed ventral hernia, No. (%)			0.81			0.0.59
<50 cm^2^	7 (9.7)	8 (12.3)		7 (12.7)	5 (9.1)	
≥50 and <100cm^2^	48 (66.7)	44 (67.7)		34 (61.8)	39 (70.9)	
≥100 cm^2^	17 (23.6)	13 (20.0)		14 (25.5)	11 (20.0)	
Etiology, No. (%)			0.48			0.61
Trauma	49 (68.1)	43 (66.2)		35 (63.6)	37 (67.3)	
Unexplained perforation	2 (2.8)	1 (1.5)		2 (3.6)	1 (1.8)	
Adhesive obstruction	14 (19.4)	18 (27.7)		13 (23.6)	15 (27.3)	
Mesenteric thrombosis	7 (9.7)	3 (4.2)		5 (9.1)	2 (3.6)	
**Characteristics related to DS**						
Hemoglobin before DS, No. (%)			0.81			0.65
<120 g/L	19 (26.4)	16 (24.6)		12 (21.8)	14 (25.5)	
≥120 g/L	53 (73.6)	49 (75.4)		43 (78.2)	41 (74.5)	
Albumin before DS, No. (%)			0.56			0.84
<35 g/L	20 (27.8)	21 (32.3)		17 (30.9)	18 (32.7)	
≥35 g/L	52 (72.2)	44 (67.7)		38 (69.1)	37 (67.3)	
Grade of abdominal adhesion, No. (%)			0.17			0.83
≤ IV	28 (38.9)	18 (27.7)		16 (29.1)	15 (27.3)	
V	47 (65.3)	44 (67.7)		39 (79.9)	40 (72.3)	
Duration of DS, No. (%)			0.15			0.84
<4 h	33 (45.8)	22 (33.8)		21 (38.2)	20 (36.4)	
≥4 h	39 (54.2)	43 (66.2)		34 (61.8)	35 (63.6)	
Bleeding loss during DS, No. (%)			0.65			0.57
<1,000 ml	12 (16.7)	9 (13.8)		8 (14.5)	6 (10.9)	
≥1,000 ml	60 (83.3)	56 (86.2)		47 (85.5)	49 (89.1)	
The amount of red blood cell suspension input during DS and 48 h after DS^*^, No. (%)			0.57			0.69
<10 U	30 (41.7)	24 (36.9)		18 (32.8)	20 (36.4)	
≥10 U	42 (58.3)	41 (63.1)		37 (67.3)	35 (63.6)	
The amount of albumin input during DS and 48 h after DS^**^, No. (%)			0.15			0.84
<100 g	32 (44.4)	21 (32.3)		20 (36.4)	21 (38.2)	
≥100 g	40 (55.6)	44 (67.9)		35 (63.6)	34 (61.8)	

* In order to maintain the Hemoglobin >100 g/L within 48 h after definitive surgery.

** In order to maintain the Albumin >30 g/L within 48 h after definitive surgery.

A total of 110 patients were further divided into PSM polymeric formula group (*n* = 55) and PSM predigested formula group (*n* = 55). Difference between the groups in the time period was observed after PSM (Table 1).

### Duration of usage of polymeric formula

The median duration of usage of polymeric formula was 4 months (IQR: 3–4 months), while the usage of EN was 5 months (IQR: 4–6 months). Before using the polymeric formula, the predigested formula was used for transition in 56 patients, and the duration of using predigested formula was 1 month (IQR: 1–2 months). There were other 16 patients without transition, and the polymeric formula was used directly.

### Postoperative ileus

Before PSM, postoperative ileus occurred in a total of 61 patients (44.5%) with 27 (37.5%) in the polymeric formula group and 34 (52.3%) in the predigested formula group (*P* = 0.04). The adjusted logistics regression indicated that the polymeric formula could result in a reduction in postoperative ileus (OR = 0.47; 95% CI: 0.21–0.95; *P* = 0.04; Table 2). After PSM, there were a total of 47 of the 110 patients with postoperative ileus, 18 (32.7%) in the polymeric formula group and 29 (52.7%) in the predigested formula group (*P* = 0.03). The polymeric formula reduced the incidence of postoperative ileus (OR = 0.42; 95% CI: 0.19–0.92; *P* = 0.03; Table 3).

**Table 2:** Logistics regression analysis of the risk factors for defecation before PSM.

**Clinical variables**	**Unadjusted regression**			**Adjusted regression**		
	**OR**	**95% CI**	***P***	**OR**	**95% CI**	***P***
Female	0.69	0.35–1.38	0.30			
Polymeric formulas	0.49	0.24–0.96	0.04	0.47	0.21–0.95	0.04
Age	1.02	0.99–1.05	0.25			
BMI	0.94	0.73–1.20	0.61			
Time period						
Year 2005–Year 2008	Ref					
Year 2008–Year 2011	0.93	0.35–2.46	0.88			
Year 2012–Year 2015	0.53	0.19–1.45	0.21			
Year 2016–Year 2019	0.53	0.20–1.37	0.19			
Interval from fistula occurred to admission	0.99	0.93–1.07	0.97			
Distance from treitz ligament to fistula						
<100 cm	Ref					
From 100 to 200 cm	0.79	0.38–2.09	0.56			
>200 cm	0.525	0.20–1.28	0.18			
Length of small intestine						
<300 cm	Ref					
≥300 cm	1.93	0.86–4.37	0.11			
Duration of CR	1.11	0.67–2.19	0.21			
Duration of enteral nutrition	1.81	0.72–3.47	0.36			
Hight output	1.25	0.67–2.33	0.49			
The area of planed ventral hernia						
<50 cm^2^	Ref					
≥50 and <100 cm^2^	1.47	0.46–4.65	0.51			
≥100 cm^2^	2.00	0.55–7.27	0.29			
Etiology, No. (%)						
Trauma	Ref					
Unexplained perforation	0.68	0.06–7.76	0.76			
Adhesive obstruction	1.54	0.69–3.46	0.29			
Mesenteric thrombosis	0.34	0.07–1.69	0.19			
Hemoglobin before DS						
<120 g/L	Ref					
≥120 g/L	0.54	0.25–1.18	0.12			
Albumin before DS						
<35 g/L	Ref					
≥35 g/L	0.83	0.39–1.73	0.61			
Grade of abdominal adhesion						
≤ IV	Ref			Ref		
V	1.94	0.92–4.06	0.08	0.62	0.44–0.88	0.009
Duration of DS						
<4 h	Ref					
≥4 h	0.66	0.33–1.32	0.244			
Bleeding loss during DS						
<1,000 ml	Ref					
≥1,000 ml	2.10	0.76–5.80	0.15			
The amount of red blood cell suspension input during DS and 48 h after DS^*^						
<10 U	Ref			Ref		
≥10 U	2.23	1.09–4.57	0.03	1.67	0.82–4.12	0.18
The amount of albumin input during DS and 48 hours after DS^**^						
<100 g	Ref			Ref		
≥100 g	2.43	1.17–5.01	0.02	1.88	0.75–4.89	0.27

* In order to maintain the Hemoglobin >100 g/L within 48 h after definitive surgery.

** In order to maintain the Albumin >30 g/L within 48 h after definitive surgery.

**Table 3:** Logistics regression analysis of the risk factors for postoperative ileus after PSM.

**Clinical variables**	**Unadjusted regression**			**Adjusted regression**		
	**OR**	**95% CI**	***P***	**OR**	**95% CI**	***P***
Female	0.94	0.67–1.32	0.72			
Polymeric formulas	0.44	0.20–0.95	0.03	0.42	0.19–0.92	0.03
Age	0.99	0.97–1.00	0.13			
BMI	0.97	0.75–1.26	0.82			
Time period						
Year 2005–Year 2008	Ref					
Year 2008–Year 2011	1.23	0.43–3.56	0.70			
Year 2012–Year 2015	0.56	0.18–1.73	0.32			
Year 2016–Year 2019	0.43	0.14–1.28	0.13			
Interval from fistula occurred to admission	0.97	0.94–1.00	0.14			
Distance from treitz ligament to fistula						
<100 cm	Ref					
From 100 to 200 cm	1.27	0.87–1.85	0.23			
>200 cm	1.02	0.78–2.36	0.46			
Length of small intestine						
<300 cm	Ref					
≥300 cm	0.39	0.28–1.65	0.68			
Duration of CR	1.11	0.67–2.19	0.21			
Duration of enteral nutrition	1.58	0.87–3.09	0.31			
Hight output	1.16	0.58–2.33	0.67			
The area of planed ventral hernia						
<50 cm^2^	Ref					
≥50 and <100 cm^2^	1.39	0.38–5.06	0.61			
≥100 cm^2^	2.17	0.51–9.09	0.29			
Etiology, No. (%)						
Trauma	Ref					
Unexplained perforation	0.74	0.06–8.56	0.81			
Adhesive obstruction	1.71	0.71–4.12	0.23			
Mesenteric thrombosis	0.59	0.11–3.27	0.55			
Hemoglobin before DS						
<120 g/L	Ref					
≥120 g/L	1.03	0.71–1.49	0.89			
Albumin before DS						
<35 g/L	Ref					
≥35 g/L	0.84	0.37–1.88	0.67			
Grade of abdominal adhesion						
≤ IV	Ref					
V	1.86	0.77–4.43	0.17			
Duration of DS						
<4 h	Ref					
≥4 h	1.41	0.31–1.47	0.61			
Bleeding loss during DS						
<1,000 ml	Ref					
≥1,000 ml	1.66	0.74–3.79	0.22			
The amount of red blood cell suspension input during DS and 48 h after DS^*^						
<10 U	Ref			Ref		
≥10 U	2.78	1.17–6.55	0.02	1.87	0.62–4.14	0.38
The amount of albumin input during DS and 48 hours after DS^**^						
<100 g	Ref			Ref		
≥100 g	2.33	1.03–5.31	0.04	1.75	0.77–3.99	0.18

* In order to maintain the Hemoglobin >100 g/L within 48 h after definitive surgery.

** In order to maintain the Albumin >30 g/L within 48 h after definitive surgery.

### Defecation time

**Figure 2:**
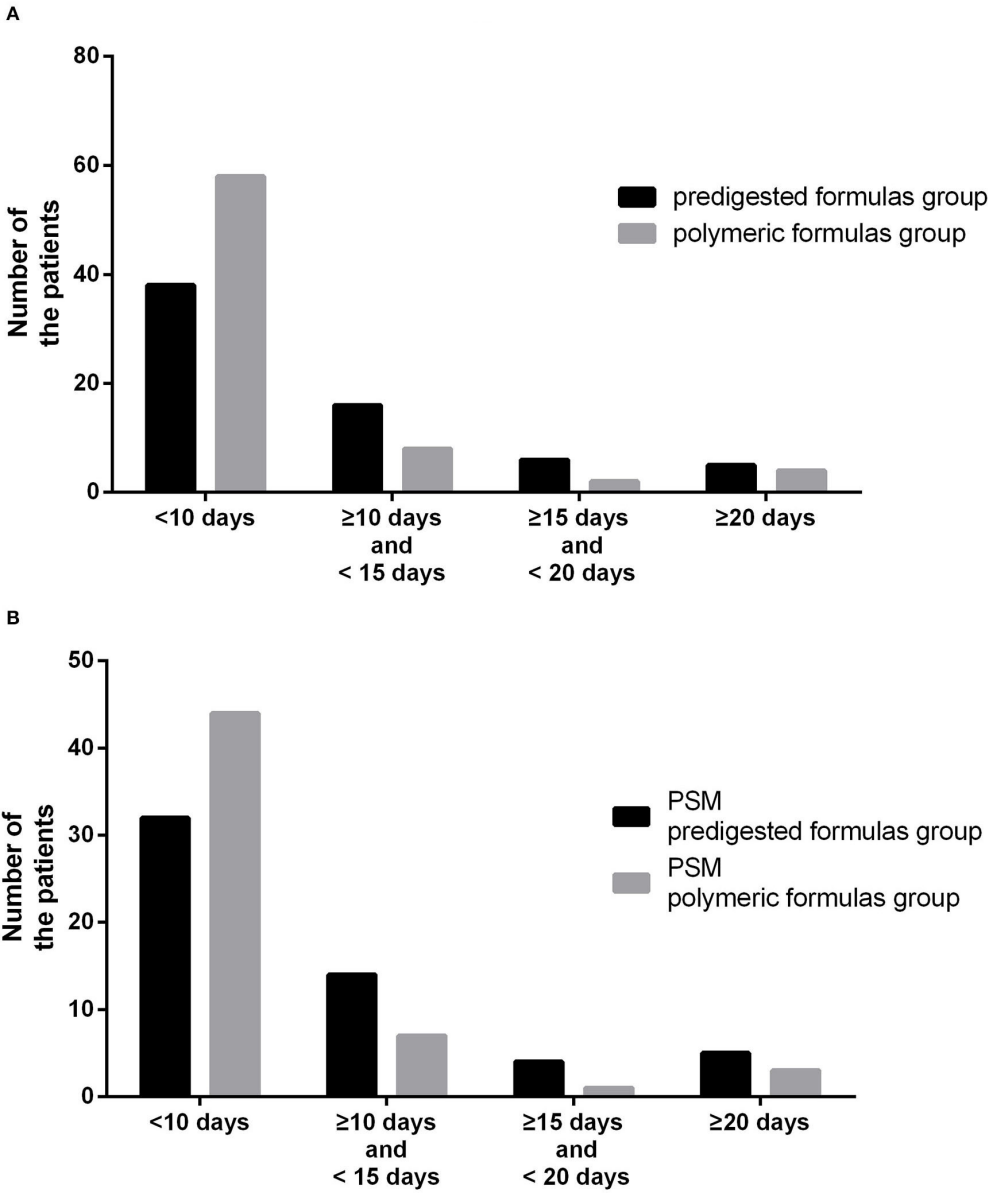
**(A)** Number of patients with different defecation times between the predigested formula group and polymeric formula group. **(B)** Number of patients with different defecation times between the PSM predigested formula group and PSM polymeric formula group.

**Figure 3:**
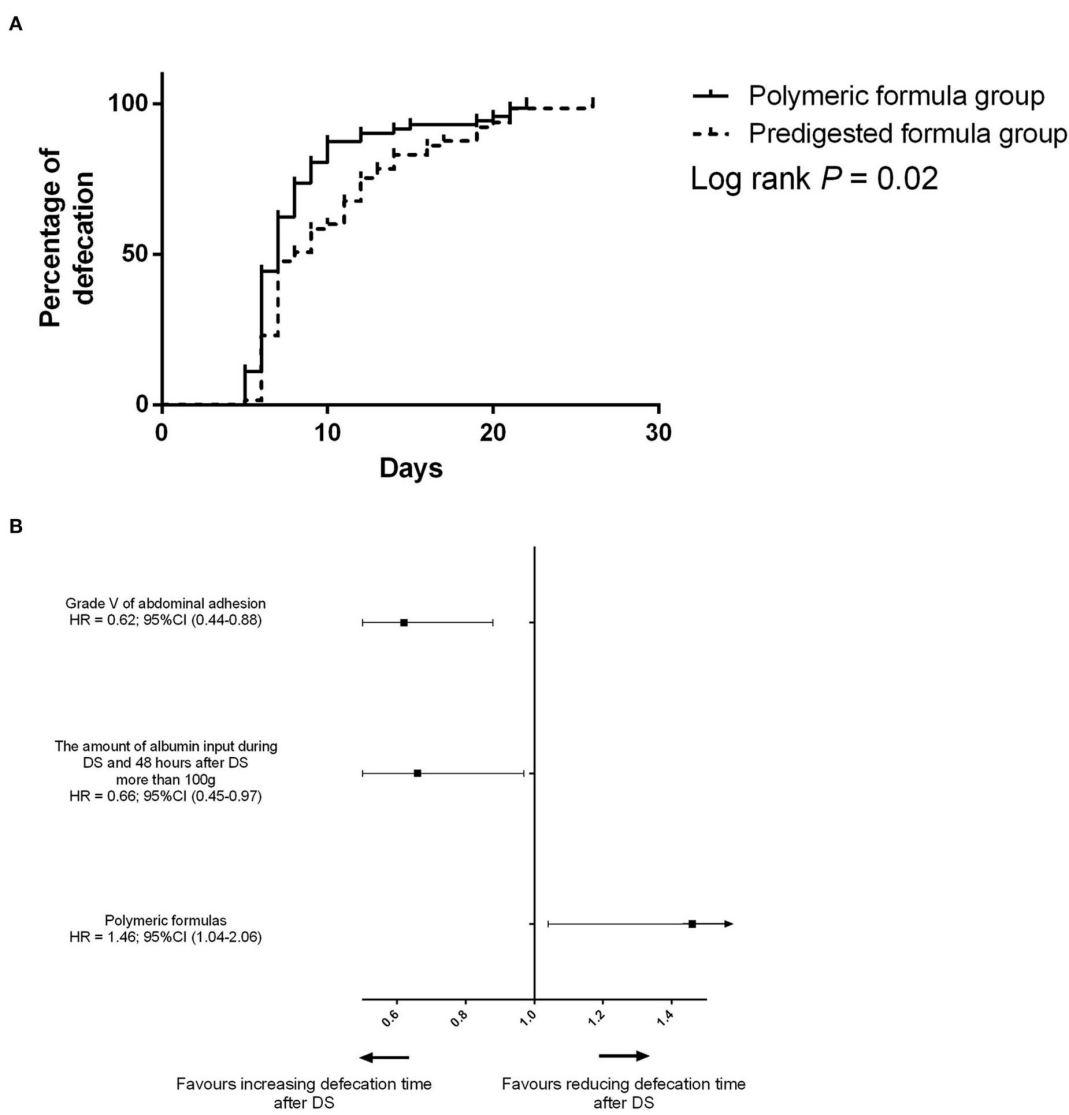
**(A)** Difference of the defecation time between the predigested formula group and polymeric formula group. **(B)** Risk factors for defecation before PSM.

Before PSM, the defecation time was 7 days (IQR: 6–11 days) in the polymeric formula group and 8 days (IQR: 7–13 days) in the predigested formula group (*P* = 0.02). In the predigested formula group, after DS, there were 27 (41.5%) patients with defecation time more than 10 days, and 5 (7.7%) patients with defecation time more than 20 days. There were 14 (19.4%) patients with defecation time more than 10 days and 4 (5.6%) patients with defecation time more than 20 days (Figure 2A) in the polymeric formula group. The preoperative usage of polymeric formula accelerated postoperative defecation (adjusted HR = 1.72; 95%CI: 1.17–2.26; *P* = 0.03, Figures 3A,B).

**Figure 4:**
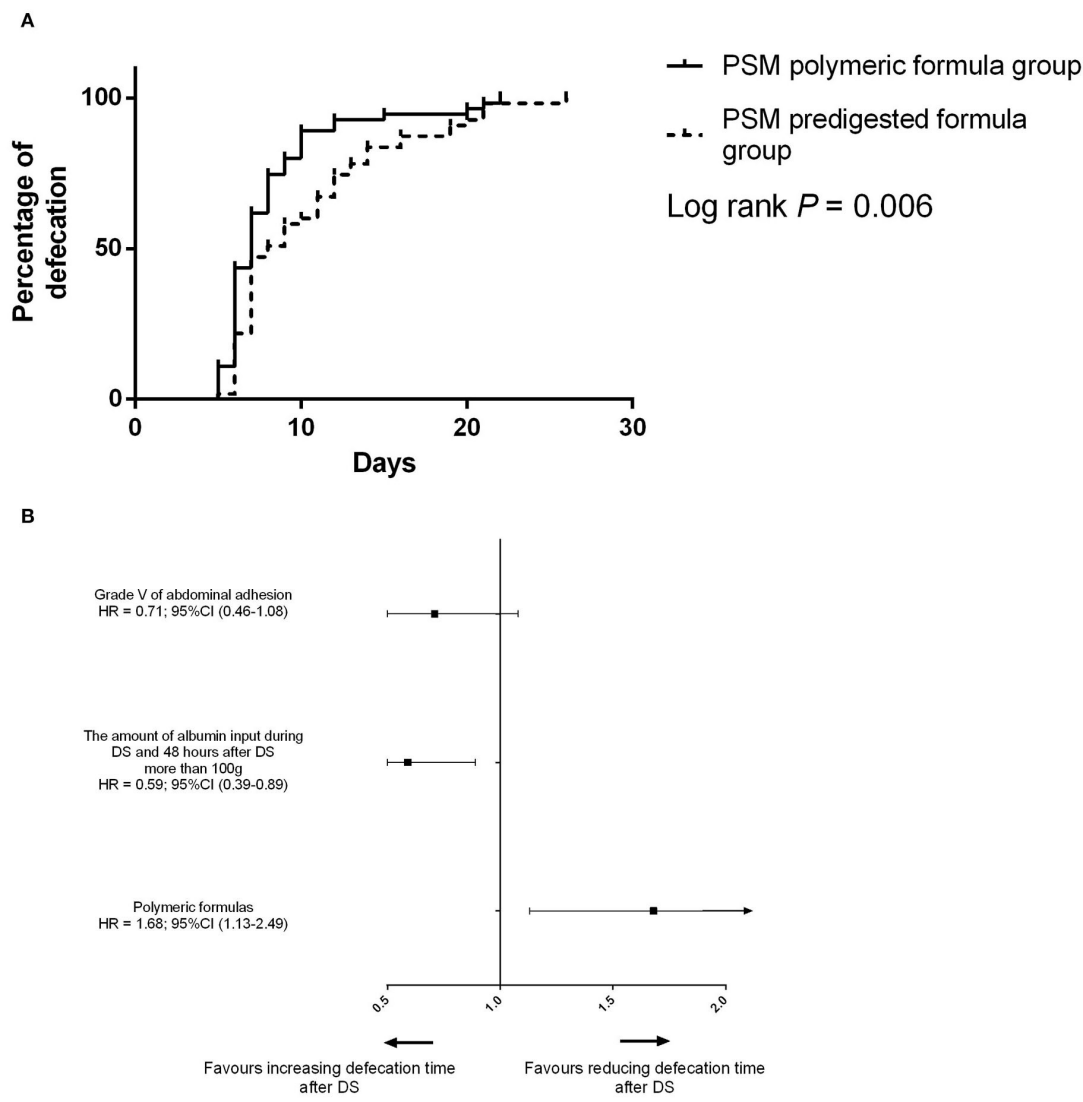
**(A)** Difference of the defecation time between the PSM predigested formula group and PSM polymeric formula group. **(B)** Risk factors for defecation after PSM.

After PSM, the defecation time was 7 days (IQR: 6–11 days) in the PSM polymeric formula group and 8 days (IQR: 7–13 days) in the PSM predigested formula group (*P* = 0.02). In the PSM predigested formula group, after DS, there were 23 (41.8%) patients with defecation time more than 10 days and 5 (9.0%) patients with defecation time more than 20 days. There were 11 (20.0%) patients with defecation time more than 10 days and 4 (7.3%) patients with defecation time more than 20 days (Figure 2B) in the PSM polymeric formula group. The preoperative usage of polymeric formula accelerated postoperative defecation (adjusted HR = 1.68; 95%CI: 1.13–2.49; *P* = 0.01, Figures 4A,B).

### The resumption from minimal EN to total EN

**Figure 5:**
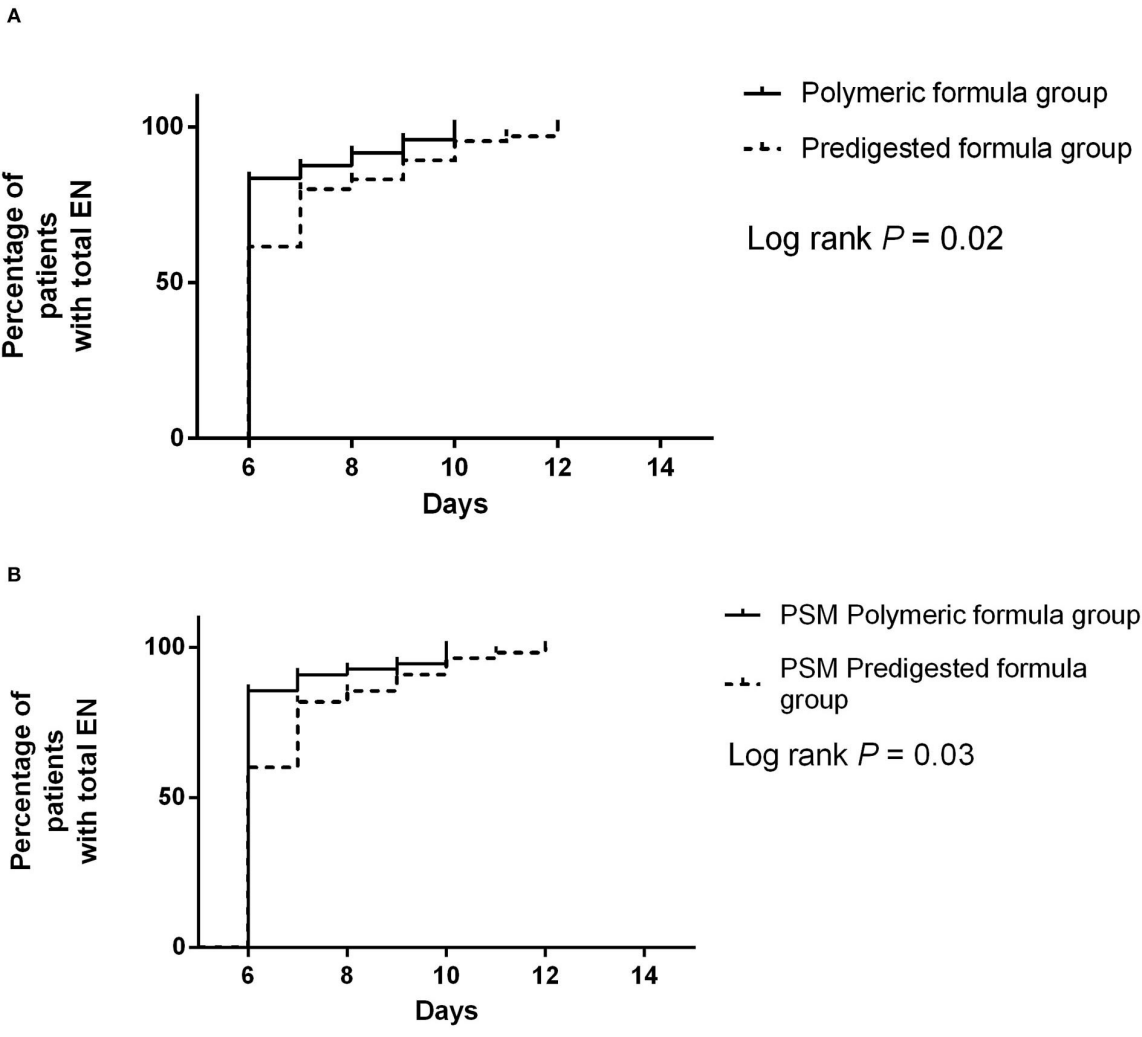
**(A)** Difference of the resumption from minimal EN to total EN between the predigested formula group and polymeric formula group. **(B)** Difference of the resumption from minimal EN to total EN between the PSM predigested formula group and PSM polymeric formula group.

Before PSM, the median duration of resumption from minimal EN to total EN in the polymeric formula group was 6 days (IQR: 6–6 days), and it was 6 days (IQR: 6–7 days) in the predigested formula group (Figure 5A). Polymeric formula accelerated the resumption process (adjusted HR = 1.32 95%CI: 1.03–3.49; *P* = 0.02). The median duration of resumption from minimal EN to total EN in the PSM polymeric formula group was 6 days (IQR: 6–6 days), and it was 6 days (IQR: 6–7 days) in the PSM predigested formula group (Figure 5B). Polymeric formula also accelerated the resumption process (adjusted HR = 1.44 95%CI: 1.02–3.17.; *P* = 0.02) after PSM.

## Discussion

In the present study, we found that postoperative ileus occurred in 45% of patients after DS for EAF, and the preoperative EN with polymeric formula appears to shorten the postoperative defecation time and reduce the incidence of postoperative ileus, although this issue has not been reported by other researchers.

The high incidence of postoperative ileus in patients with EAF was not a particularly innovative discovery. In a previous study including 159 subjects, it was similarly demonstrated that the overall incidence of postoperative ileus after DS for EAF was more than 50% (6). During the DS, severe abdominal adhesion could lead to extensive bowel and substantial blood loss. Those adverse intraoperative events not only exacerbate the postoperative inflammation response (14, 15) but also inhibit adrenergic neural pathways (15), leading to a high incidence of postoperative ileus.

While ensuring effective CR, the present study has identified that the polymeric formula may minimize the possibility of postoperative ileus compared with predigested formula. Predigested formula is more straightforward and readier assimilated and has enhanced the tolerance. As a result, it has been reported to present nutritional and clinical benefits in nutritionally high-risk non-ICU patients suffering from intestinal failure (16). In patients with EAF, most intestinal juice leaks from the fistula and cannot flow into the distal small intestine of the fistula, resulting in excessive output (5, 17–19). Accordingly, as the predigested formula needs little further intraluminal digestion and is easily absorbed (9), it has been extensively utilized in EAF patients. However, with the same energy supplement, it can be inferred that the use of predigested formula may result in more nutrients being absorbed in the proximal small intestine and lead to decreased nutrient concentration in the distal ileum.

The replacement of gastrointestinal epithelium occurs within a short period, usually between 3 and 6 days (20). This replacement requires the delivery of adequate nutrients, and gastrointestinal epithelial cells must receive their nutritional requirements intraluminally to guarantee replacement. Villous length and crypt depth are decreased due to mucosal atrophy caused by a lack of luminal nutrients (9). These pathophysiologic processes might result in intestinal absorption and mobility insufficiency, contributing to the development of postoperative ileus following intestinal surgery (21). Compared to polymeric formula, the predigested formula may reduce the nourishing effect of chyme in the digestive tract on the distal ileum, which eventually produces the potential incidence of postoperative ileus.

The problem of postoperative ileus caused by gastrointestinal disuse has been previously reported. Most of those studies focused on the complete disuse of the distal gastrointestinal tract after the temporary stoma (21–24). Unlike these studies, the present study is mainly concerned with the influence of relative disuse on gastrointestinal motility, following different types of EN formula. However, it was revealed that even with the same energy density, the difference in nutrient composition might lead to the relative disuse of distal ileum owing to the difference in absorption and digestion, which then might subsequently contribute to the varied incidence of postoperative ileus.

Our study's protocol of postoperative nutritional support was different from the current feeding strategy for gastrointestinal surgery consisting of early EN or oral administration. Surgery for EAF always has a high incidence of complications resulting from the sizeable peeling surface, the large amount of oozing blood during the surgery, and the severe postoperative intestinal edema. Oral or EN after defecation is an ancient strategy, but at least it seems safe. So, we still use this strategy when a complicated enterocutaneous fistula surgery is performed. In our study, our procedure for achieving postoperative EN feeding objectives is divided into two phases. The first component is the postoperative defecation time, whereas the second is the postoperative resumption of EN. Indeed, while there was a statistically significant difference in the length from the first postoperative restart of EN, this difference is unlikely to be clinically meaningful given that the median period for both groups was 6 days, with Figures 5A,B demonstrating a slight difference. However, when the defection time following surgery and the fraction of patients in various defecation phases were evaluated, it seemed that intervention with different components of EN solution had some impact on bowel function recovery.

Our research has certain limitations. First, the retrospective nature and the small sample size of this research might account for the deviation. A further randomized controlled study was welcome. However, to our knowledge, EAF is relatively rare, and the number of patients enrolled in our study might be the most so far. In addition, there seemed to be few studies focused on the association between the different types of EN formula and postoperative ileus. The second limitation was that in our study, besides the different nitrogen sources, there are also disparities between the content of fat and saccharides in the two nutrient formulas. As a result, a further study employing a module diet might better distinguish which nutrients play a crucial role in gastrointestinal mucosal nutrition. The third limitation was that the morphological data of terminal ileal cells cannot be collected completely in our investigation. Consequently, comparing the morphology of terminal ileal mucosa in patients with different nutrient formulas was challenging. In fact, during the DS, when anastomosis was performed after the fistula was excised, we observed that, in the predigested formula group, the closer the fistula is to the terminal ileum, the smaller the diameter of the bowel (not shown in our study). However, this phenomenon is not apparent in the polymeric formula group. Additionally, the majority of patients in the polymeric formula group also received the predigested formula as a transition. It might result in a diminished impact of polymeric formula on reducing the postoperative ileus. Another limitation was that a possible bias existed introduced by the fact that the study cohorts were at differing time periods.

## Conclusion

This study has demonstrated that compared with predigested formula, the preoperative polymeric formula appears to be associated with earlier recovery of bowel function after DS for EAF.

## Data availability statement

The original contributions presented in the study are included in the article/supplementary material, further inquiries can be directed to the corresponding author/s.

## Ethics statement

The studies involving human participants were reviewed and approved by Jinling Hospital Ethics Committee. The patients/participants provided their written informed consent to participate in this study.

## Author contributions

RZ and WT provide research objects. XX and RZ collected and analyzed the data. ZY, XX, and WT wrote the main manuscript text. XX prepared figures and revised the manuscript. ZY designed the research. SL organized data. All authors contributed to the article and approved the submitted version.

## Conflict of interest

The authors declare that the research was conducted in the absence of any commercial or financial relationships that could be construed as a potential conflict of interest.

## Publisher's note

All claims expressed in this article are solely those of the authors and do not necessarily represent those of their affiliated organizations, or those of the publisher, the editors and the reviewers. Any product that may be evaluated in this article, or claim that may be made by its manufacturer, is not guaranteed or endorsed by the publisher.
